# Safety and Efficacy of Contrast Media Administration via Selected Vascular Access Devices in Computed Tomography

**DOI:** 10.3390/jcm15134958

**Published:** 2026-06-25

**Authors:** Damian Romańczuk, Sandra Lange, Wioletta Mędrzycka-Dąbrowska, Grzegorz Cichowlas

**Affiliations:** 1 Department of Anesthesiology and Intensive Care, Medical University of Gdańsk, 80-952 Gdańsk, Poland; damian.romanczuk@gumed.edu.pl; 2 Department of Anesthesiology and Intensive Care, University Clinical Center, 80-952 Gdańsk, Poland; 3 Department of Internal and Pediatric Nursing, Medical University of Gdańsk, 80-211 Gdańsk, Poland; langa94@gumed.edu.pl; 4 Department of Anesthesiology and Intensive Care Education, Medical University of Warsaw, 02-097 Warsaw, Poland; grzegorz.cichowlas@wum.edu.pl; 5 Department of Anesthesiology and Intensive Care, Czerniakowski Hospital sp. z.o.o., 00-739 Warsaw, Poland

**Keywords:** computed tomography (CT), contrast media, vascular access devices (VAD), power injector, contrast extravasation, peripheral intravenous cannula (PIVC), totally implantable venous access device (TIVAD), diffuser technology, patient safety, high-pressure injection

## Abstract

**Background**: The administration of intravenous contrast media using automated power injectors is fundamental for high-quality computed tomography (CT), particularly in CT angiography (CTA). The selection of an appropriate vascular access device (VAD) and adherence to technical safety standards are critical for ensuring diagnostic efficacy and patient safety. This systematic review aims to synthesize current scientific literature regarding the efficacy and safety of contrast media infusion across various vascular access routes, including peripheral intravenous cannulas (PIVC), central venous catheters (CVC), and totally implantable venous access devices (TIVAD). **Methods**: The review followed PRISMA 2020 guidelines. A comprehensive search was conducted in PubMed, CINAHL, Web of Science, and Scopus for studies published between 2000 and 2026. A total of 19 studies—including randomized controlled trials (RCTs), cohort studies, and systematic reviews—were analyzed. Methodological quality was assessed using Joanna Briggs Institute (JBI) appraisal tools. **Results**: Modern PIVCs utilizing diffuser technology (side holes) significantly reduce distal jet pressure, minimizing vessel wall damage during high-flow injections. For patients with difficult vascular access, “power-injectable” certified devices (e.g., PICCs or TIVADs) serve as a safe alternative. Standard, non-power-injectable central lines must be avoided due to the risk of catheter rupture. The selection of an appropriate vascular access device is particularly challenging in patients of older age or those with chronic conditions, such as peripheral vascular disease, obesity, or chemotherapy-related venous damage, which often lead to difficult intravenous access (DIVA). **Conclusions**: Utilizing certified power-injectable devices and advanced cannula designs improves the safety of high-pressure contrast administration. Adherence to technical protocols and the identification of high-risk patients are essential for mitigating complications such as contrast extravasation.

## 1. Introduction

Intravenous vascular access established for contrast medium administration in computed tomography (CT) is crucial for acquiring high-quality contrast-enhanced scans and is indispensable for angiographic examinations. Currently, the majority of imaging protocols utilize an automated power injector for intravenous contrast delivery. This allows for the administration of the contrast medium as a continuous bolus at high flow rates, which is essential for advanced procedures, including multiphase organ imaging and CT angiography (CTA) [1,2]. The use of an automated power injector yields greater reproducibility of bolus hemodynamic parameters compared to manual contrast injection, which has been associated with significant pressure variability [3]. In addition to ensuring optimal timing for the enhancement of anatomical structures, a major advantage of automated injection systems is reducing ionizing radiation exposure to radiology staff [1,3].

Current safety standards dictate that to prevent severe complications, such as an air embolism or contrast extravasation, the examination must be performed by qualified medical personnel utilizing appropriate techniques and up-to-date knowledge of vascular access devices [2,3].

### 1.1. Background

Contrast medium extravasation remains one of the most concerning complications associated with power-injected CT. Due to the hypertonicity of the agent, its displacement into the extravascular space can trigger a localized inflammatory response, potentially leading to tissue necrosis or ulceration. In severe cases, particularly involving large volumes of contrast, the development of compartment syndrome may occur, necessitating urgent surgical consultation and potentially life-altering interventions such as fasciotomy [3,4,5].

**Figure 1:**
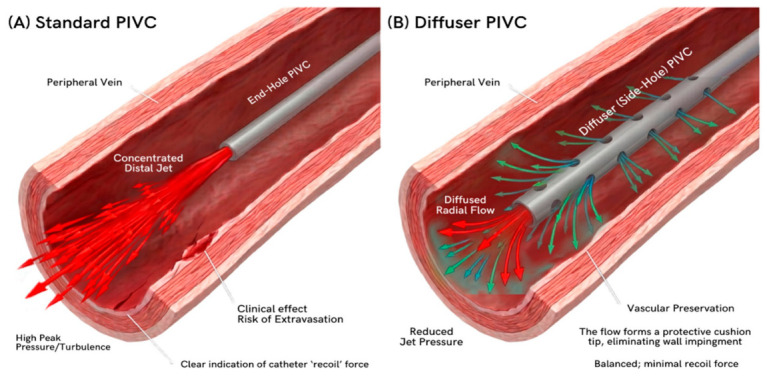
Comparison of contrast medium infusion dynamics between standard and diffuser peripheral intravenous cannulas (PIVC). (**A**) Standard PIVC with an end-hole design, demonstrating a concentrated distal jet, higher peak pressure, and increased turbulence, which may elevate the risk of extravasation. (**B**) Diffuser PIVC with side-holes, demonstrating diffused radial flow, reduced jet pressure, and optimal vascular preservation. (The figure was generated using an AI-based tool, Gemini 3.5 Flash Google LLC, Mountain View, CA, USA).

Identifying patients at high risk for such vascular complications is essential. Factors such as advanced age, impaired consciousness, or compromised venous integrity due to chronic conditions (e.g., peripheral vascular disease, prior chemotherapy) significantly increase the likelihood of access failure. In standard scenarios, the preferred access route remains a peripheral intravenous cannula (PIVC) inserted in the antecubital fossa or the forearm. Modern cannula designs featuring side holes (diffuser technology) enable a significant reduction in the distal pressure of the contrast jet, thereby minimizing the risk of vessel wall damage during high-flow injections (Figure 1) [1,2,4].

However, in patients with fragile veins (characterized as difficult vascular access), the placement of a central venous catheter prevents the need for multiple peripheral cannulation attempts. This is particularly applicable to intensive care unit (ICU) patients, as well as individuals undergoing long-term chemotherapy or dialysis therapy [1,2]. To ensure patient safety, facilitate precise interdisciplinary communication, and standardize clinical procedures regarding these various access routes on a global scale, the classification of vascular access devices (VADs) adopted in this study follows the latest international standards. A key reference point is the NAVIGATE project—a joint consensus of the global organizations GloVANet (Global Vascular Access Network) and WoCoVA (World Conference on Vascular Access) published by van Rens et al. (2025) [6].

**Figure 2:**
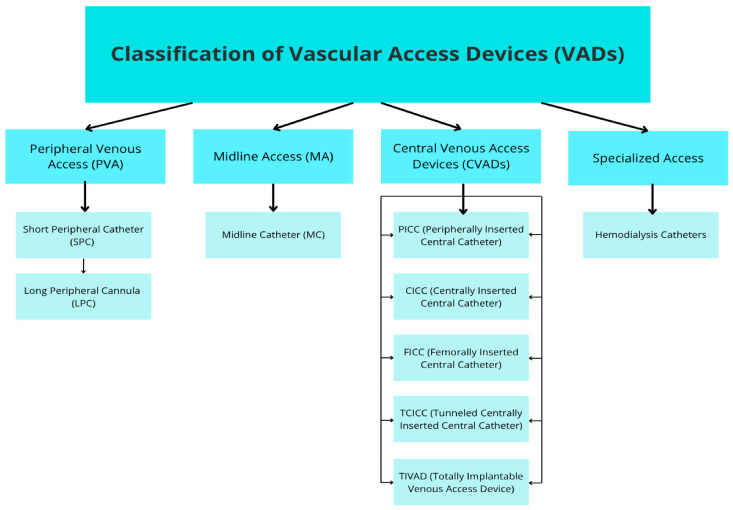
Classification of Vascular Access Devices (VADs) [6].

In the context of computed tomography, these devices are broadly classified not only by their anatomical placement (peripheral vs. central) but crucially by their pressure tolerance. Only devices specifically certified and clearly labeled for high-pressure injections—termed “power-injectable”—can be safely used for automated contrast administration. While standard PIVCs are utilized for routine scans with gauge sizes dictating the maximum permissible flow rates, alternative power-injectable options like peripherally inserted central catheters (PICCs) and totally implantable venous access devices (TIVADs, commonly known as vascular ports) are utilized when peripheral veins are inadequate. Standard, non-power-injectable central lines and ports must never be used with automated injectors due to the severe risk of catheter rupture or fragmentation. The detailed classification of contrast-compatible vascular access devices, outlining their specific categories and pressure designations, is presented in Figure 2.

Despite the existence of previous publications, there remains a significant gap in clinical practice, as evidenced by the continued occurrence of errors and complications during high-pressure contrast administration. Furthermore, the body of high-quality, up-to-date research specifically addressing the safety of newer vascular access models remains disproportionately small compared to the rapidly evolving technology of power injectors. Given that patient safety and the occupational health of medical personnel are of paramount importance, a comprehensive and updated systematic review is essential. This work aims to bridge the gap between theoretical guidelines and clinical reality, providing a consolidated framework for minimizing risks and optimizing diagnostic efficacy.

### 1.2. Aim

The aim of this review is to provide a comprehensive synthesis of current scientific literature regarding the efficacy and safety of contrast media infusion depending on the vascular access utilized. The analysis specifically focuses on evaluating the flow performance of peripheral cannulas, assessing the safety of central lines during automated injection, and identifying critical risk factors and protective measures associated with contrast extravasation.

The research questions were:

Q1: How do the caliber and specific design features of peripheral intravenous cannulas (e.g., diffuser technology) influence flow performance and the frequency of high-pressure alarms during automated contrast delivery?
Q2: What is the clinical safety profile of utilizing central venous catheters (CVCs) and vascular ports for high-pressure contrast media injection, specifically regarding device integrity and patency?
Q3: What are the primary clinical and procedural risk factors associated with contrast medium extravasation, and which protective factors or protocols are most effective in mitigating this complication?

## 2. Materials and Methods

### 2.1. Study Design and Guidelines

This systematic review was prepared and reported in accordance with the current PRISMA 2020 (Preferred Reporting Items for Systematic Reviews and Meta-Analyses) [7] guidelines to ensure high transparency and full reproducibility of the source identification and selection process. The process of systematic data collection and critical bibliographic analysis was conducted between February and March 2026 (Supplementary Materials).

### 2.2. Search Strategy and Screening Process

To gather the research material, a comprehensive search of three electronic medical and scientific databases was conducted: PubMed, Web of Science, and Scopus. The search strategy was based on a combination of controlled Medical Subject Headings (MeSH) terms and specialized keywords, connected by the Boolean operators AND and OR.

In accordance with the aim of the review, which is to analyze the correlation between the type of access and the safety of infusion, the strategy was expanded to include terms related to technical complications, such as high-pressure alarms, catheter displacement, and vascular access damage. Following the initial search, duplicate records were removed. The screening process was conducted in two stages: first, titles and abstracts were independently evaluated by two reviewers to determine their relevance; subsequently, the full texts of potentially eligible articles were assessed against the predefined criteria. Any discrepancies between the reviewers were resolved through discussion or consultation with a third reviewer.

The search strategy in the PubMed database was refined using specific filters to ensure the relevance and quality of the retrieved records. To ensure full reproducibility and explain the specific search yield, it should be clarified that the following inclusion criteria were applied directly within the database as automated filters: article types (Randomized Controlled Trial, Clinical Controlled Trial, Review, and Systematic Review), text availability (Full text), and age group (Adult: 19+ years). The application of these rigorous pre-search filters explains the retrieval of the final 12 records from the initially broader pool of results.

Although the search was open to both English and Polish languages, none of the Polish-language records met the final inclusion criteria; therefore, 100% of the articles ultimately included in this review are in English. Detailed search strings for each database are documented in Table 1 and Table 2. The inclusion and exclusion criteria for studies in the review are presented in Table 3.

### 2.3. Data Extraction

The data extraction process was conducted independently by two reviewers using a standardized data extraction form to ensure the reliability and consistency of the analysis. Detailed information was extracted from each eligible study, including the first author’s name, publication year, study design, sample size, and clinical patient profile, with a particular focus on inpatient and outpatient populations. As part of the intervention characteristics, data regarding the type of vascular access device utilized were collected, including peripheral cannulas (incorporating diffuser technology), PICCs, midline catheters, as well as central venous catheters (CVCs) and vascular ports with power-injectable certification. The analysis of technical parameters encompassed the contrast medium flow rate, catheter diameter, and the use of automated power injectors. Primary and secondary endpoints focused on infusion safety, including the incidence of extravasations, high-pressure alarms, line patency, and the risk of vascular access displacement or damage. Any discrepancies between the reviewers during the data collection phase were resolved through consensus or via consultation with a third member of the research team.

### 2.4. Data Synthesis

A quantitative meta-analysis was not conducted due to the substantial clinical and methodological heterogeneity observed across the included literature. Clinical heterogeneity was profound, driven by the wide variation in the evaluated vascular access devices (ranging from standard peripheral cannulas to complex central venous catheters and TIVADs of differing French sizes and lumen designs), diverse contrast media properties (e.g., varying iodine concentrations, viscosities, and warming practices), and disparate institutional injection protocols (with flow rates ranging from low to high-pressure injections > 5 mL/s). Furthermore, significant methodological heterogeneity was present. The included papers encompassed a broad spectrum of study designs, including retrospective cohorts, prospective observational studies, and randomized trials. Crucially, the operational definitions and detection methods for primary clinical endpoints, such as extravasation, were highly inconsistent across studies—varying from automated power-injector pressure alarms to direct visual clinical observation or delayed patient reporting. Given this extensive heterogeneity, pooling the data into a single meta-analytic mathematical model would yield statistically unreliable and clinically misleading effect sizes.

Consequently, a structured narrative synthesis was employed, facilitating a multifaceted mapping of the scientific evidence. The results were grouped thematically around the primary research questions concerning infusion safety, device technical parameters, and clinical risk factors. Data derived from primary studies and conclusions from previous systematic reviews were integrated descriptively and supplemented with summary tables. This approach enabled a robust evaluation of the efficacy of various vascular access types (peripheral and central) and the identification of gaps in the current medical literature regarding the high-pressure administration of contrast media.

To mitigate the risk of evidence overlap and potential double counting arising from the inclusion of both primary studies and previous systematic reviews, a careful methodological approach was adopted. Importantly, because a quantitative meta-analysis was not performed, the statistical double counting of patient populations was inherently avoided. Instead, a complementary narrative synthesis was employed. Previous systematic reviews (e.g., Ding et al., Behzadi et al., Buijs et al. [1,8,9]) were utilized strictly to establish overarching clinical safety profiles and broad epidemiological baseline risk factors for extravasation. Concurrently, the included primary studies were analyzed without overlapping extraction to provide updated evidence on recent technological advancements—such as diffuser technology in peripheral cannulas and specific high-pressure parameters of modern TIVADs—which were not adequately covered in the earlier reviews.

### 2.5. Quality Assessment

The methodological quality of the included studies was independently evaluated by two reviewers using the Joanna Briggs Institute (JBI) critical appraisal tools. Depending on the study design, specific instruments were applied, including the JBI Checklist for Randomized Controlled Trials (Table 4), JBI Critical Appraisal Checklist for Cohort Studies (Table 5), the JBI Checklist for Systematic Reviews and Research Syntheses (Table 6), the JBI Checklist for Case Series (Table 7), and the JBI Checklist for Quasi-Experimental Studies (Table 8). Study quality was classified into three categories: high (≥80%), moderate (≥65%), and low (≤55%), with a point awarded only when the response to a specific criterion was “Yes”. Any disagreements between reviewers were resolved through discussion until a consensus was reached or by consultation with a third member of the research team.

## 3. Results

### 3.1. Study Selection

**Figure 3:**
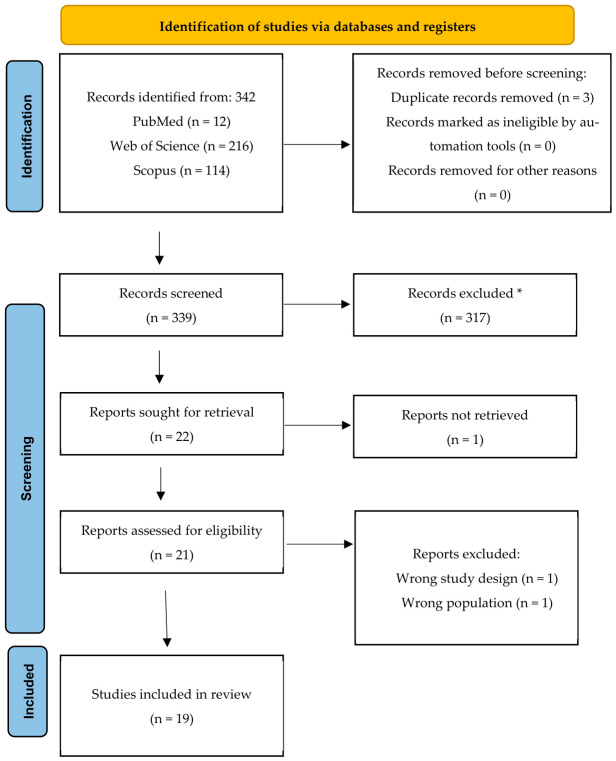
PRISMA 2020 flow diagram of study selection. Consider, if feasible to do so, reporting the number of records identified from each database or register searched (rather than the total number across all databases/registers). * If automation tools were used, indicate how many records were excluded by a human and how many were excluded by automation tools (Supplementary Materials, PRISMA 2020 Checklist).

The evidence selection process was conducted in multiple stages, strictly adhering to the methodological rigor of the PRISMA 2020 guidelines [7]. The initial search of PubMed, Web of Science, and Scopus databases yielded 342 records (12 from PubMed, 216 from Web of Science, and 114 from Scopus). Prior to screening, 3 duplicate records were identified and removed. Two independent reviewers screened 339 records based on titles and abstracts, evaluating congruence with objectives and inclusion criteria (2000–2026, adult population, automated injectors). Records not meeting these criteria were excluded, leaving a subset for full-text evaluation. This rigorous analysis confirmed the suitability of the articles for addressing research questions. As a result, 18 studies were qualified for qualitative synthesis: 3 from PubMed, 11 from Web of Science, and 4 from Scopus (Figure 3).

### 3.2. Characteristics of the Included Studies

The study selection process, conducted in accordance with the strict methodology of the PRISMA 2020 guidelines, resulted in the final inclusion of 19 scientific publications. The studies included in the review demonstrate significant heterogeneity in terms of study design, reflecting the complexity of vascular access issues in diagnostic imaging. The analyzed material encompasses randomized controlled trials (RCTs) [5 studies], prospective and retrospective cohort studies [5 studies], case series [5 studies], quasi-experimental studies [1 study], and previous systematic reviews [3 studies]. The methodological quality of all included studies was rigorously assessed using the Joanna Briggs Institute (JBI) critical appraisal tools, with the majority demonstrating high or moderate reliability. To ensure a rigorous synthesis and avoid evidence overlap, findings derived from observational studies and previous systematic reviews were primarily used to establish epidemiological baselines, whereas data from RCTs and prospective trials were prioritized to evaluate the efficacy and safety of specific, newer vascular access technologies.

The analyzed population consisted of adult inpatients and outpatients who required contrast-enhanced computed tomography (CT), including advanced procedures such as CT angiography (CTA) or multiphase imaging. The intervention characteristics focused on the use of automated contrast injectors, which facilitate the administration of the contrast medium as a continuous bolus at high flow rates. The evaluated vascular access devices (VADs) included standard peripheral intravenous cannulas (PIVCs), modern diffuser-type cannulas equipped with side holes, midline catheters, peripherally inserted central catheters (PICCs), central venous catheters (CVCs), and vascular ports (totally implantable venous access devices—TIVADs).

Key technical parameters extracted included the contrast medium flow rate, catheter diameter, and their power-injectable certification. The primary endpoints of the analyzed studies focused on infusion safety, with particular emphasis on the incidence of contrast extravasation and air embolism. Additionally, operational parameters were evaluated, such as the occurrence of high-pressure alarms during injector operation, the maintenance of vascular line patency, and the risk of mechanical damage, catheter rupture, or dislodgement during the procedure. The synthesis of the collected data facilitated a multidimensional assessment of the efficacy of various vascular access types under the conditions of power-injected contrast media administration (Table 9).

### 3.3. Flow Performance in Relation to Peripheral Cannula Caliber and Design

A prominent theme across the included studies is the optimization of flow dynamics based on the design and gauge of peripheral intravenous cannulas (PIVCs). While large-bore cannulas (e.g., 18 G) have traditionally been recommended to accommodate high flow rates, recent observational evidence indicates that smaller calibers with modernized designs may facilitate equally effective contrast administration [12,27]. Multiple studies suggest a potential advantage of side-hole (fenestrated or diffuser) cannulas over traditional non-fenestrated models [12,14]. The incorporation of side holes has been reported to reduce both intravascular resistance and recoil force, thereby supporting the safer application of higher flow rates. Notably, even small-caliber (24 G) fenestrated cannulas have been described as potentially safe and effective alternatives to standard 22 G single-end-hole cannulas in MDCT examinations within specific patient cohorts [13]. In cases of difficult venous access, an alternative approach utilizing two simultaneous small peripheral cannulas has been proposed as a feasible method to achieve the cumulative high injection rates required for diagnostic imaging [29].

### 3.4. Safety Profiles of Central Venous Catheters (CVCs) and Vascular Ports

The use of central venous access devices for power injections presents a clinical profile that is primarily dependent on the specific device type. Available observational literature suggests that standard central venous catheters (CVCs) can be utilized for rapid contrast injection, provided that specific institutional protocols and flow limits are strictly observed [2,11]. Within the realm of totally implantable vascular access devices (TIVADs), several studies highlight the clinical utility of power-injectable ports, which appear to provide adequate contrast enhancement while maintaining an acceptable safety profile compared to peripheral access in oncology patients [18,19,20,24,26]. However, comparative analyses emphasize that complication rates may vary significantly between modern power-injectable systems and standard, non-rated ports [17]. This underscores the clinical importance of verifying the port’s pressure rating prior to contrast administration. Additionally, specific mechanical complications remain a concern with central lines; for instance, the phenomenon of catheter tip “flipping” (displacement or reversal) has been documented in peripherally inserted central catheters (PICCs) subjected to automated power injections [25].

### 3.5. Risk Factors and Extravasation Prevention

The phenomenon of contrast media extravasation is influenced by a multifactorial matrix of patient-related, mechanical, and procedural variables [8,9]. Pooled data from the included systematic reviews identify patient frailty, older age, and compromised vessel integrity as frequently reported physiological risk factors. Beyond patient demographics, injection parameters and the method of administration are thought to play a significant role, with observable differences in extravasation incidence and mechanisms when comparing manual injections to automated power injectors [3]. Furthermore, the human factor appears to influence procedural safety. Observational data indicate a potential correlation between the incidence of extravasation and the professional background of the individual establishing the vascular access; specifically, differences in safety profiles were noted between cannulations performed by general ward nurses versus specialized radiology personnel in some settings [21]. These findings collectively suggest the importance of standardized training protocols and the routine testing of venous access patency immediately prior to the initiation of automated high-pressure injections.

## 4. Discussion

### 4.1. Summary of Main Findings

The primary objective of this comprehensive systematic review was to synthesize the correlation between vascular access types and the efficacy, hemodynamics, and safety of automated contrast media infusion. The body of evidence aggregated herein demonstrates that both peripheral and central vascular access devices (VADs) can be successfully utilized for power-injected contrast administration. However, this clinical success is not uniform; it demands a highly tailored, device-specific methodology rather than a standardized approach. The findings emphasize that mitigating the risk of complications—such as high-volume extravasation or catheter rupture—relies heavily on adherence to calibrated technical parameters (flow rates, pressure limits) and an understanding of device design.

### 4.2. Flow Performance in Relation to Peripheral Cannula Caliber and Design

Historically, standard clinical practice in diagnostic imaging often dictated that large-bore peripheral cannulas (e.g., 18-gauge) were preferred for high-flow contrast administration. However, the evidence synthesized in this review provides new perspectives on this practice. A prospective randomized controlled trial conducted by Johnson et al. (2014) [27] served as an important reference, indicating that modern, smaller-caliber 20-gauge fenestrated cannulas—engineered with diffuser technology—are capable of achieving flow rates of 5.0 mL/s with comparable reliability to traditional 18 G models. The incorporation of side holes alters the fluid dynamics of the contrast jet, which may reduce peak pressure at the catheter tip. This design adaptation is associated with a high first-pass cannulation success rate, reduced endothelial trauma, and a low incidence of recorded extravasation events.

Recent evidence from Gavin et al. (2023) [15] demonstrates that perforated peripheral catheters provide optimal contrast flow rates in oncology patients with a safety profile comparable to standard devices. These findings strongly support the use of diffuser technology for vascular preservation.

Furthermore, managing patients with Difficult Venous Access (DVA) requires alternative strategies. In a clinical observation, Son et al. (2020) [24] proposed a method for this specific population. Their study documented high efficacy and safety by simultaneously utilizing two small cannulas (22 G and 24 G) via a Y-connector to achieve diagnostic flow parameters (3.0–4.0 mL/s). This dual-access approach provided sufficient image enhancement without subjecting compromised vessels to high-pressure loads, offering a potential alternative for personalized vascular management.

### 4.3. Safety Profile of Central Venous Catheters (CVCs) and Vascular Ports

In specific patient cohorts, such as intensive care and oncology patients, the utilization of existing central venous catheters (CVCs) and totally implantable vascular ports represents a practical alternative to repeated peripheral venipuncture. Despite historical caution regarding central line integrity during power injection, the gathered evidence suggests a high feasibility and acceptable safety profile when institutional protocols are employed. Research by Herts et al. (2001) [11] reported a 94% procedural success rate for high-pressure injections through diverse central catheters (including triple-lumen Hickman and Leonard designs), without recorded instances of catheter rupture.

These findings were evaluated on a larger scale in a systematic review by Buijs et al. (2017) [1]. Encompassing a dataset of over 1000 automated injections, their analysis revealed that while a small percentage of minor catheter dislodgement occurred (ranging from 2.2% to 15.4%), complete catheter rupture was not observed. Nonetheless, utilizing standard CVCs for contrast delivery involves specific adjustments: current protocols often require downregulating injector pressure limits (e.g., 50–190 psi, compared to the 300 psi threshold standard for peripheral access) and restricting injection rates to 1.5–3.3 mL/s. While this pressure-gating ensures mechanical integrity, it may occasionally result in a marginally delayed or attenuated enhancement profile in major structures like the aorta or pulmonary arteries.

In oncology, totally implantable vascular ports are frequently used for longitudinal patient monitoring. The cohort study by Plumhans et al. (2012) [17] provided evidence that power-injectable ports do not carry a significantly higher risk of long-term complications—such as thrombosis, deep-seated infection, or tip migration—when compared to standard, non-injectable ports. These devices offer a reliable conduit for routine CT surveillance, potentially addressing the challenge of vascular exhaustion in cancer patients.

A crucial consideration during routine CT surveillance is the generation of beam-hardening artifacts, which are prominent with entirely titanium ports and can obscure adjacent anatomy. Conversely, ports made of plastic polymers or composite materials generate markedly fewer artifacts. To optimize image quality, clinicians should preferentially select or upgrade to plastic or composite models for patients requiring frequent CT scans.

To address port-related CT artifacts, advanced reconstruction algorithms can be highly effective. Specifically, virtual monoenergetic images (VMI) derived from dual-energy spectral-detector CT have been shown to significantly reduce these artifacts. Utilizing high-energy VMI—particularly in the 140–200 keV range—substantially diminishes both hypo- and hyperdense artifacts around the port chamber and catheter. This algorithmic approach decreases image noise and significantly improves the diagnostic assessment of surrounding soft tissues, such as the pectoral and subclavian regions [30].

The impact of port-related metal artifacts is also a notable consideration in positron emission tomography/computed tomography (PET/CT) hybrid imaging. Standard reconstructions often result in prominent bright and dark band artifacts on the CT component. To mitigate this, iterative metal artifact reduction (iMAR) algorithms can be employed. As demonstrated in a prospective study by Martin et al. [31], applying various iMAR algorithms significantly reduces these CT artifacts in the vicinity of the port chamber, thereby improving morphological assessment. Interestingly, however, their study revealed that despite the substantial improvement in CT image quality (measured by Hounsfield units), there were no significant differences in PET attenuation correction or consecutive standardized uptake value (SUV) measurements. Therefore, while iMAR is highly beneficial for anatomical CT evaluation, routine PET quantification appears robust even without specialized artifact reduction [32,33].

### 4.4. Risk of Extravasation and Protective Factors

While the overall safety profile of contrast administration is high, the risk of extravasation remains a critical clinical consideration during automated infusion. Current literature highlights that the prevention of this complication is closely linked to specific clinical practices. For peripheral lines, the low incidence of extravasation in studies utilizing fenestrated cannulas [27] suggests that diffuser technology acts as a protective factor by reducing localized vascular wall stress.

The safety of high-flow rates has been particularly elucidated by a prospective study conducted by Wienbeck et al. (2010) [34], involving a cohort of over 4400 patients. The authors demonstrated that the use of automated injectors at flow rates of 5–8 mL/s is a safe procedure, with an overall extravasation rate of only 1.2%, showing no significant correlation with either the flow rate itself or the contrast medium concentration. This study provides evidence that the risk of complications is more closely related to anatomical factors (e.g., hand dorsum vs. antecubital fossa location) and physical cannula parameters (smaller diameters, such as 22 G, were associated with higher complication rates) than to the pressure generated by the injector. These findings support the necessity of individualizing the selection of puncture site and cannula gauge as primary determinants of safety, regardless of the high-speed infusion technology applied.

Conversely, when utilizing central venous catheters (CVCs) for contrast delivery, strict adherence to pressure-gating and manufacturer-specified flow limits are essential protective factors against vessel or catheter damage.

## 5. Study Limitations and Methodological Heterogeneity

Despite the systematic approach of this synthesis, the interpretation of these findings must be contextualized within the limitations of significant methodological heterogeneity inherent to the available literature. A primary limitation of this review is the diversity of the incorporated study architectures—ranging from retrospective case series to prospective RCTs and systematic reviews. While the inclusion of both primary studies and previous systematic reviews introduces a potential risk of narrative evidence overlap, this was addressed by using earlier reviews primarily to establish a broad epidemiological baseline, while primary studies were analyzed to evaluate technical advancements not covered previously.

Furthermore, disparities in institutional injection protocols, varying contrast media viscosities, and inconsistent definitions in complication-reporting systems (e.g., automated pressure alarms versus clinical observation of extravasation) precluded the execution of a quantitative meta-analysis. Consequently, while the majority of the selected papers achieved high or moderate reliability scores upon evaluation with the Joanna Briggs Institute (JBI) critical appraisal tools, the variability in defined clinical endpoints necessitates a degree of scientific caution. These findings should serve as a foundational reference rather than universal, inflexible clinical mandates. This study also incorporates the pivotal prospective analysis by Wienbeck et al., which, despite being excluded from standard search processes due to a highly unique and targeted selection of keywords, represents one of the most critical evaluations of high-flow safety (up to 8 mL/s) in diagnostic imaging. Its inclusion is essential for a comprehensive understanding of the correlation between vascular access techniques and the risk of extravasation [34].

## 6. Conclusions and Directions for Future Research

In light of the synthesized data, the contemporary landscape of radiological vascular access supports an individualized approach. The selection of an access route for power-injected contrast should ideally be tailored to both the patient’s vascular integrity and the specific biomechanical characteristics of the equipment. Utilizing modern, fenestrated peripheral cannulas represents a reasonable strategy that may optimize peripheral safety without compromising imaging quality. Concurrently, the use of CVCs and power-injectable ports—while associated with an acceptable safety profile when utilized appropriately—requires careful attention to established pressure limits and flow dynamics. Ultimately, this review highlights the value of updating interdisciplinary training protocols and suggests a need for future prospective, multicenter trials to further clarify the interplay between advanced medical devices and human vascular physiology.

## 7. Implications for Practice

The findings of this systematic review suggest practical considerations that can be considered within diagnostic imaging departments and hospital wards to optimize patient safety. Based on the synthesized evidence, the following tentative points are proposed for clinical consideration:

- Individualized Assessment: The choice of vascular access should be based on an individual evaluation of the patient’s venous status, rather than historical standard routines.
- Consideration of Newer Designs: When using peripheral access, the implementation of fenestrated or diffuser-type cannulas may be considered as a means to potentially reduce local vascular pressure and contrast extravasation risks.
- Verification of Central Device Ratings: Prior to utilizing central venous lines or totally implantable ports, verifying the manufacturer’s pressure and flow rate specifications is essential to support mechanical safety.
- Protocol Adherence: For non-power-injectable central lines, adhering strictly to modified pressure-gated protocols (e.g., reducing pressure limits and flow rates) represents an important safety measure when peripheral access is unavailable.

## 8. Key Recommendations for Clinical Practice Include

- Revision of Peripheral Cannula Selection Criteria

Medical personnel should transition away from the routine use of large-bore cannulas (e.g., 18 G) in favor of smaller sizes (20 G and 22 G), with a preference for fenestrated (side-hole) catheters. The selection of a smaller cannula size is particularly recommended for patients with difficult venous access (DVA), as well as oncology and geriatric patients, as it significantly reduces vessel trauma while safely sustaining flow rates of 3–5 mL/s.

- Standardization of Vascular Port and Central Venous Catheter Management
  - The use of totally implantable venous access ports (TIVAPs) and central venous catheters (CVCs) for automated power injections necessitates the strict implementation of rigorous verification protocols. The imaging team must:
  - Always confirm (based on patient records, implant cards, or radiopaque markers) that the specific port and catheter are certified as power-injectable.
  - Exclusively use dedicated, power-injectable Huber needles (in the case of ports).
  - Consistently assess system patency prior to connecting the automated injector by performing blood aspiration and dynamic flushing with 0.9% NaCl, which rules out mechanical catheter occlusion.
- Optimization of Automated Injector Settings

Radiologic technologists and nurses operating automated infusion pumps must strictly utilize the pressure limit monitoring function. Setting the pressure limit (typically at 300 psi for standard power-injectable access devices) ensures the immediate cessation of the injection in the event of intravascular resistance, serving as a critical barrier against catheter rupture or massive extravasation.

- Standardization of Medical Staff Training

Given the proven correlation between complication rates and the location where the vascular access was established (hospital ward vs. radiology department), the implementation of interdisciplinary training programs is recommended. Ward nurses responsible for peripheral venous cannulation in patients referred for CT scans should undergo periodic training regarding the specific requirements of power injections. This education should encompass the optimal selection of the insertion site—prioritizing the forearm and, as a second choice, the antecubital fossa, based on the best clinical outcome for the patient—and the proper securing of the cannula to prevent dislodgement.

- Early Response Protocol for Extravasation

Radiology departments must possess an updated management algorithm for contrast media extravasation. Close observation of the insertion site during the initial seconds of bolus administration remains a fundamental duty of the staff, as even the most advanced pressure monitoring systems cannot substitute the clinical assessment of developing tissue edema.

## Figures and Tables

**Table 1:** Search strategies.

Databases	Search Strategy
PubMed	(“contrast media”[MeSH Terms] OR “contrast media”[TiAb] OR “power injection”[TiAb]) AND (“vascular access devices”[MeSH Terms] OR “catheters”[MeSH Terms] OR “peripheral intravenous catheter”[TiAb] OR “midline”[TiAb] OR “PICC”[TiAb] OR “central venous catheter”[TiAb] OR “implantable port”[TiAb] OR “diffuser”[TiAb]) AND (“safety”[TiAb] OR “extravasation”[TiAb] OR “complications”[TiAb] OR “pressure alarm”[TiAb] OR “catheter displacement”[TiAb] OR “catheter rupture”[TiAb]) Limit: Filters applied: Year 2000–2026, Polish and English Language, article types (Randomized Controlled Trial, Clinical Controlled Trial, Review, and Systematic Review), text availability (Full text), and age group (Adult: 19+ years) Results: 12
Web of Science	TS = ((“contrast media” OR “contrast agent” OR “power injection”) AND (“vascular access” OR catheter* OR cannula* OR midline OR PICC OR port*) AND (safety OR extravasation OR complication* OR “high pressure” OR rupture)) Limit: Filters applied: Year 2000–2026, Polish and English Language Results: 216
Scopus	TITLE-ABS-KEY ((“contrast media” OR “contrast agent” OR “power inject*”) AND (“vascular access” OR catheter* OR cannula* OR midline OR PICC OR port) AND (“computed tomography” OR ct OR angiography) AND (“pressure alarm*” OR “flow rate” OR “pressure limit” OR fenestrated OR diffuser OR extravasation)) Limit: Filters applied: Year 2000–2026, Polish and English Language Results: 114

**Table 2:** PICO framework.

	Inclusion Criteria
Population (P)	Adult patients (inpatients and outpatients) requiring a computed tomography (CT) examination with the use of an intravenous contrast medium.
Intervention (I)	Automated high-pressure infusion of contrast medium (utilizing an automated power injector). Administration of the medium as a continuous bolus at high flow rates for diagnostic purposes (CT angiography, multiphase imaging).
Comparison (C)	Comparison of various types of vascular access devices (VADs): Peripheral cannulas (standard vs. diffuser technology). Midline catheters and PICCs. Central venous catheters (CVCs) and vascular ports with power-injectable certification.
Outcome (O)	Safety and efficacy of infusion: Incidence and types of complications (extravasations, air embolisms). Technical parameters (high-pressure alarms, line patency). Access integrity (risk of displacement, damage, or catheter rupture).

**Table 3:** Inclusion and exclusion criteria.

	Inclusion Criteria	Exclusion Criteria
Publication type	Primary studies (prospective, retrospective, observational), RCTs, systematic reviews, and meta-analyses.	Case reports, letters to the editor, editorials, and conference abstracts.
Population	Adult patients undergoing CT examinations (angiography, multiphase imaging).	Studies on animal models or phantoms.
Intervention	Administration of contrast medium using an automated power injector (high-pressure).	Exclusively manual contrast administration (with the exception of comparison groups).
Type of access	Peripheral cannulas (standard and diffuser types), PICCs, Midline catheters, CVCs, and vascular ports (power-injectable).	Vascular access devices lacking high-pressure certification (unless this is the subject of the complication analysis).
Outcomes	Extravasations, high-pressure alarms, catheter displacement or damage, line patency.	Allergic reactions, contrast-induced nephropathy (unrelated to the vascular access placement technique).
Databases	PubMed, CINAHL, Web of Science, Scopus	Other databases, Gray literature,
Language	Polish and English	Publications in languages other than Polish or English.
Timeframe	Publications from 2000 to 2026.	Published before the year 2000.

**Table 4:** JBI Critical Appraisal Tools—checklist for randomized controlled trials [10].

Author, Year	Q1	Q2	Q3	Q4	Q5	Q6	Q7	Q8	Q9	Q10	Q11	Q12	Q13	Total
Herts et al., 2001 [11]	Y	U	Y	N	N	Y	Y	U	N	Y	Y	Y	Y	8/13
Johnson et al. (2014) [12]	Y	Y	Y	N	N	Y	Y	Y	Y	Y	Y	Y	Y	11/13
Tamuta et al. 2017 [13]	U	N	Y	N	N	U	Y	Y	Y	Y	Y	Y	Y	8/13
Kim et al. 2019 [14]	Y	U	Y	N	N	Y	Y	Y	Y	Y	Y	Y	Y	10/13
Gavina et al. (2024) [15]	Y	Y	Y	N	N	Y	Y	Y	Y	Y	Y	Y	Y	11/13

Y—Yes, N—No, U—unclear; High (if ≥80% of the assessment tool items received a point), Moderate (if ≥65% of the assessment tool items received a point); Low ≤ 55% (if ≤55% of the assessment tool items received a point). Q1—Was true randomization applied for assigning participants to treatment groups? Q2—Was the allocation to treatment groups adequately concealed? Q3—Were the treatment groups comparable at baseline in terms of key characteristics? Q4—Were participants blinded to their assigned treatment group? Q5—Were the individuals administering the intervention blinded to group allocation? Q6—Were outcome assessors blinded to the participants’ group assignment? Q7—Apart from the intervention under study, were the treatment groups managed identically? Q8—Was follow-up complete, and if not, were differences in follow-up between groups appropriately described and analyzed? Q9—Were participants analyzed in the groups to which they were originally randomized? Q10—Were outcomes measured consistently across all treatment groups? Q11—Were outcome measurements performed in a valid and reliable manner? Q12—Was an appropriate statistical approach used for data analysis? Q13—Was the study design suitable, and were any deviations from the standard RCT framework (e.g., individual randomization, parallel groups) properly addressed in the conduct and analysis of the trial?

**Table 5:** JBI Critical Appraisal Checklist for Cohort Studies [16].

Author, Year	Q1	Q2	Q3	Q4	Q5	Q6	Q7	Q8	Q9	Q10	Q11	Total
Plumhans et al. (2012) [17]	Y	Y	Y	Y	Y	Y	Y	Y	U	U	Y	9/11
Washio et al. 2024 [18]	Y	Y	Y	Y	Y	Y	Y	Y	Y	U	Y	10/11
Goltz et al. 2012 [19]	Y	Y	Y	N	N	Y	Y	Y	Y	U	Y	8/11
Goltz et al. 2011 [20]	U	Y	Y	N	Y	Y	Y	Y	Y	U	Y	8/11
Kingston et al. 2012 [21]	Y	Y	Y	U	N	Y	Y	Y	Y	N	Y	8/11

Y—Yes, N—No, U—unclear; High (if ≥80% of the assessment tool items received a point), Moderate (if ≥65% of the assessment tool items received a point); Low ≤ 55% (if ≤55% of the assessment tool items received a point). Q1—Were the two groups similar and recruited from the same population? Q2—Were the exposures measured similarly to assign people to both exposed and unexposed groups? Q3—Was the exposure measured in a valid and reliable way? Q4—Were confounding factors identified? Q5—Were strategies to deal with confounding factors stated? Q6—Were the groups/participants free of the outcome at the start of the study (or at the moment of exposure)? Q7—Were the outcomes measured in a valid and reliable way? Q8—Was the follow up time reported and sufficient to be long enough for outcomes to occur? Q9—Was follow up complete, and if not, were the reasons to loss to follow up described and explored? Q10—Were strategies to address incomplete follow up utilized? Q11—Was appropriate statistical analysis used?

**Table 6:** JBI Critical Appraisal Checklist for Systematic Reviews and Research Syntheses [22].

Author, Year	Q1	Q2	Q3	Q4	Q5	Q6	Q7	Q8	Q9	Q10	Q11	Total
Buijs et al. (2017) [1]	Y	Y	Y	N	Y	Y	Y	Y	N	Y	Y	9/11
Ding et al. (2018) [8]	Y	Y	Y	Y	Y	Y	Y	Y	U	Y	Y	10/11
Behzadi et al., 2018 [9]	Y	Y	Y	Y	Y	Y	Y	Y	U	Y	U	9/11

Y—Yes, N—No, U—unclear; High (if ≥80% of the assessment tool items received a point), Moderate (if ≥65% of the assessment tool items received a point); Low ≤ 55% (if ≤55% of the assessment tool items received a point). Q1—Is the review question clearly and explicitly stated? Q2—Were the inclusion criteria appropriate for the review question? Q3—Was the search strategy appropriate? Q4—Were the sources and resources used to search for studies adequate? Q5—Were the criteria for appraising studies appropriate? Q6—Was critical appraisal conducted by two or more reviewers independently? Q7—Were there methods to minimize errors in data extraction? Q8—Were the methods used to combine studies appropriate? Q9—Was the likelihood of publication bias assessed? Q10—Were recommendations for policy and/or practice supported by the reported data? Q11—Were the specific directives for new research appropriate?

**Table 7:** JBI Critical Appraisal Checklist for Case Series [23].

Author, Year	Q1	Q2	Q3	Q4	Q5	Q6	Q7	Q8	Q9	Q10	Total
Son et al. 2020 [24]	Y	Y	Y	Y	Y	Y	Y	Y	N	Y	9/10
Sosa Lozano et al., 2012 [25]	Y	Y	Y	Y	Y	Y	Y	Y	U	Y	9/10
Sanelli et al. 2004 [2]	Y	Y	Y	Y	Y	Y	Y	Y	U	Y	9/10
Teichgräber et al. 2012 [26]	Y	Y	Y	Y	Y	Y	Y	Y	U	Y	9/10
Johnson et al., 2014 [27]	Y	Y	Y	Y	Y	Y	Y	Y	Y	Y	10/10

Y—Yes, N—No, U—unclear; High (if ≥80% of the assessment tool items received a point), Moderate (if ≥65% of the assessment tool items received a point); Low ≤ 55% (if ≤55% of the assessment tool items received a point). Q1—Were there clear criteria for inclusion in the case series? Q2—Was the condition measured in a standard, reliable way for all participants included in the case series? Q3—Were valid methods used for identification of the condition for all participants included in the case series? Q4—Did the case series have consecutive inclusion of participants? Q5—Did the case series have complete inclusion of participants? Q6—Was there clear reporting of the demographics of the participants in the study? Q7—Was there clear reporting of clinical information of the participants? Q8—Were the outcomes or follow up results of cases clearly reported? Q9—Was there clear reporting of the presenting site(s)/clinic(s) demographic information? Q10—Was statistical analysis appropriate?

**Table 8:** JBI Critical Appraisal Tools—checklist for quasi-experimental studies [28].

Author, Year	Q1	Q2	Q3	Q4	Q5	Q6	Q7	Q8	Q9	Total
Son et al. (2018) [29]	Y	N	Y	N	Y	Y	Y	Y	Y	7/9

Y—Yes, N—No. High (if ≥80% of the assessment tool items received a point); Moderate (if ≥65% of the assessment tool items received a point); Low ≤ 55% (if ≤55% of the assessment tool items received a point. Q1—Does the study clearly distinguish the “cause” from the “effect,” ensuring there is no ambiguity about which variable occurs first? Q2—Were the participants included in the comparisons comparable in key characteristics? Q3—Did participants in the comparison groups receive similar care or treatment, aside from the intervention or exposure under investigation? Q4—Was there a control group included in the study? Q5—Were outcome measures collected at multiple time points, both before and after the intervention or exposure? Q6—Was follow-up complete, and if not, were any differences in follow-up between groups adequately described and analyzed? Q7—Were the outcomes of participants in the comparison groups measured in a consistent manner? Q8—Were the outcome measurements conducted in a reliable and valid way? Q9—Was an appropriate statistical analysis applied to the data?

**Table 9:** Synthesis of Included Studies.

First Author, Year	Study Design	Study Group (n)	Country	VAD Type	Size (G/Fr)	Injection Rate (mL/s)	Injection Pressure	Outcomes
Buijs S.B. et al., 2017 [1]	Systematic Review	n = 7 studies included (Totaling over 1000+ contrast injections via CVCs)	The Netherlands	Central Venous Catheters (CVCs)	Various (Standard clinical CVC sizes used in the included studies)	1.5–3.3 mL/s	Limits: 50–190 psi	No catheter ruptures reported across all 7 studies. Catheter dislocation rate: 2.2–15.4%. Contrast enhancement was sufficient for diagnostic purposes, though sometimes lower in pulmonary arteries compared to PIV.
Herts B.R. et al., 2001 [11]	Prospective observational study	n = 225 patients (295 CT scans total); Study group: 174 (CVC), Control: 51 (PIV)	USA	CVCs: Implantable ports (Bardport), Hickman (triple-lumen), Leonard (double-lumen), Arrow (triple-lumen)	Ports/Leonard: 9.6 Fr; Hickman: 10 Fr; Arrow: 7 Fr (16 G/18 G lumens); Huber needles: 19 G	1.5–2.5 mL/s (CVC group); 2.0–3.0 mL/s (PIV group)	Limit: 100 psi (for CVC); 300 psi (for PIV). Manufacturers’ rating: 15–25 psi	Feasibility: 94%. Safety: No catheter ruptures/malfunctions. Efficacy: Slightly lower hepatic/aortic enhancement vs. PIV due to flow rate limits.
Plumhans C. et al., 2012 [17]	Prospective cohort study	n = 94 oncology patients (High-pressure port group: 49; Standard port group: 45)	Germany	Totally implantable access ports: Power-injectable (Xcela, Navilyst Medical) vs. Standard (Vortex, AngioDynamics	8 Fr	Up to 5.0 mL/s	Limit: 300 psi	No significant difference in implantation success or complication rates (thrombosis, infection, catheter tip migration) between high-pressure and standard ports. High-pressure ports are safe and reliable for CT follow-up.
Johnson P.T. et al., 2014 [12]	Prospective Randomized Controlled Trial	n = 200 patients (Randomized study: 100 with 18 G standard vs. 100 with 20 G fenestrated)	USA	Peripheral IV catheters (PIV): Standard nonfenestrated vs. Fenestrated (diffuser tip—BD Nexiva Diffusics)	18 G (Standard) vs. 20 G (Fenestrated/Diffuser)	5.0 mL/s	Peak pressure recorded; Limit set for high-flow MDCT (Dual Source 128-MDCT)	20 G fenestrated catheters achieved 5 mL/s flow as reliably as 18 G standard. Reduced peak pressure and significantly higher first-attempt success rate for 20 G. No extravasations in either group.
Son B.G. et al., 2018 [29]	Prospective clinical study	n = 68 patients	South Korea	Two small peripheral IV catheters	22 G and 24 G	3.0–4.0 mL/s	Limit: 300 psi	Feasibility: 100%. Successful high-rate injection in all patients. No extravasation or catheter-related complications. Image quality (hepatic enhancement) was comparable to standard protocols with large-bore catheters.
Ding S. et al., 2018 [8]	Systematic Review and Meta-analysis	n = 25 studies included	Switzerland	Peripheral IV catheters	18 G to 22 G	Various; analyzed as a risk factor for extravasation	High-pressure power injection protocols	Overall extravasation rate: 0.1% to 0.9%. Risk factors identified: use of automated power injectors, female sex, and non-radiology staff performing cannulation.
Behzadi A.H. et al., 2017 [9]	Systematic Review	n = 1,104,872 patients (Meta-analysis of 17 studies; 2191 extravasations)	USA	Peripheral IV catheters	Various	High flow rates identified as a risk factor for extravasation	Use of automated power injectors	Extravasation rate: 0.26% for CT (6× higher than for MRI). Key risk factors: female gender, older age, in-patients, existing IV lines (instead of new), and failure to warm contrast media to body temperature.
Washio H. et al., 2024 [18]	Retrospective cohort study	n = 217 patients (CV delay: 72, CV routine: 74, Peripheral Access: 71)	Japan	Central Venous (CV) Ports vs. Peripheral Intravenous Access (PA)	Ports: Power-injectable; Peripheral: 20 G or 22 G catheters	1.3–2.5 mL/s	Limit set to 300 psi for power-injectable ports	No extravasation or catheter-related complications in any group. Enhancement in the portal venous phase was comparable between CV ports and peripheral access, provided that a short delay (approx. 4 s) was added for ports to account for the longer travel time of contrast.
Son R.S. et al., 2020 [24]	Retrospective multicenter study	n = 417 oncology patients	South Korea	Totally implantable venous power ports	Power-injectable Huber needles (size not specified, standard for power ports)	High-rate injection	Power injection mode	Success rate: 98.9% (534/540 scans). No major complications (no ruptures, no serious extravasations). Catheter failure was significantly associated with using high-concentration contrast media (>350 mg I/mL).
Goltz J.P. et al., 2012 [19]	Retrospective study	n = 204 patients	Germany	Totally Implantable Venous Power Ports	5.8 Fr to 8 Fr (Catheter sizes); Huber needles: 19 G	2.0–5.0 mL/s	Limit: 300 psi	Technical success: 100%. High-pressure injection was successful in all 74 performed CT scans. No catheter tip migration (displacement) was observed after power injection. Complication rate was low and comparable for both sites.
Sosa Lozano L.A. et al., 2012 [25]	Prospective observational study	n = 67 patients	USA	Power Injectable PICC	4 Fr (single lumen) and 5 Fr (double lumen)	Mean: 3.6 mL/s (Range: 0.8–5 mL/s)	Power injection mode	Displacement (flip) rate: 15.4% (12/78 cases). Catheters located above the tracheobronchial angle (TBA) flipped in 62.5% of cases. Catheters below TBA flipped only in 10%. No clinical complications (no venous wall injuries).
Tamura A. et al., 2017 [13]	Prospective Randomized Study	n = 180 patients	Japan	Peripheral IV catheters: 24 G with side-holes vs. 22 G standard end-hole	24 G (Side-hole) vs. 22 G (Standard)	2.0 mL/s	Significantly lower mean pressure in 24 G side-hole group (61.5 psi) vs. 22 G group (78.3 psi)	24 G side-hole catheters generated significantly lower injection pressure than larger 22 G catheters. No extravasations in either group. Contrast enhancement (HU) was identical in both groups.
Sanelli P.C. et al., 2004 [2]	Prospective Study	n = 15 neuro-ICU patients	USA	Standard CVC: Triple-lumen 7 Fr	7 Fr	Up to 4.0 mL/s	84–115 psi	No catheter ruptures or failures in both phases. Safe use of standard CVC for high-rate (4 mL/s) CT angiography. Successful diagnostic enhancement achieved in all cases.
Kim J. et al., 2019 [14]	Prospective Randomized Study	n = 300 patients (Group 1: 20 G standard; Group 2: 20 G fenestrated; Group 3: 22 G fenestrated)	South Korea	Peripheral IV catheters	20 G (Standard) vs. 20 G (Fenestrated) vs. 22 G (Fenestrated)	High-flow for Cardiac MDCT	Significantly lower in 20 G fenestrated (208.3 PSI) vs. 20 G standard (216.9 PSI), *p* = 0.006	Fenestrated catheters (20 G) provided significantly higher vascular attenuation (HU) in coronary arteries and aorta compared to standard 20 G. Zero extravasations in all 300 cases.
Teichgräber U.K. et al., 2012 [26]	Prospective observational study	n = 98 patients	Germany	Power-injectable central venous port systems	8 Fr and needle 19 G	2.0–4.0 mL/s	Mean peak pressure: 153.2 psi (Range: 105–216 psi)	Technical success: 100%. No complications (rupture, displacement, extravasation) during 207 power injections. High patient satisfaction (8.8/10). No occlusion or infection related to power injection use.
Johnson P.T. et al., 2010 [27]	Prospective observational study	n = 1000	USA	Peripheral IV catheters	Range: 18 G to 24 G (Most common: 20 G—56%, 22 G—34%)	Mean rates: 18 G (3.6 mL/s), 20 G (2.9 mL/s), 22 G (2.1 mL/s), 24 G (1.5 mL/s)	Recorded during clinical CT protocols	Success rate: 98%. Average of 1.2 attempts per patient. Extravasation rate: 0.3% (3/1000). Catheter gauge significantly limited the achieved flow rate, but 22 G was sufficient for most routine non-CTA scans.
Goltz J.P. et al., 2011 [20]	Retrospective study	n = 141	Germany	Conventional TIVAPs	Forearm ports (various standard gauges for upper extremity)	1.5 mL/s	Limit: 150 psi	Technical success: 100%. No catheter ruptures, failures, or extravasations. Adequate diagnostic image quality achieved. No catheter tip migration observed.
Kingston R.J. et al., 2012 [21]	Prospective observational study	n = 31,101	Australia	Peripheral IV catheters	Various (Standard hospital range: 18 G to 22 G)	High-rate power injection (standard CT protocols)	Recorded via automated power injectors	Overall extravasation rate: 0.12% (38/31,101). Risk was significantly higher for ward staff cannulations (0.23%) compared to radiology staff cannulations (0.09%). Most extravasations occurred in the hand or wrist rather than the antecubital fossa.
Gavin N.C. et al., 2024 [15]	Randomized Controlled Trial	n = 101	Australia	Peripheral: Standard vs. Perforated (Nexiva Diffusics)	20 G & 22 G	High-rate (≥3 mL/s)	N/A (protocol)	1 failure (extravasation) in intervention group. Comparable enhancement & satisfaction.

## Data Availability

The data on which our review is based are available in the manuscripts of the included articles.

## Supplementary Materials

The following supporting information can be downloaded at: https://www.mdpi.com/article/10.3390/jcm15134958/s1, PRISMA 2020 Checklist.
